# The global landscape of lean metabolic dysfunction-associated steatotic liver disease: insight from Asia and the West

**DOI:** 10.3389/fgstr.2025.1699508

**Published:** 2025-12-02

**Authors:** Hery Djagat Purnomo, Randy Adiwinata, Cecilia Oktaria Permatadewi, Hesti Triwahyu Hutami, Didik Indiarso

**Affiliations:** Division of Gastroentero-Hepatology, Department of Internal Medicine, Dr Kariadi Hospital, Faculty of Medicine, Diponegoro University, Semarang, Indonesia

**Keywords:** metabolic dysfunction-associated steatotic liver disease, Asia, West, fatty liver, genetic polymorphism

## Abstract

Metabolic dysfunction-associated steatotic liver disease (MASLD) has emerged as a leading global cause of chronic liver disease, affecting 25–30% of the population. While MASLD is traditionally associated with obesity, lean MASLD—a subset characterized by hepatic steatosis and metabolic dysfunction in individuals with a normal body mass index (BMI)—is increasingly recognized as a distinct clinical entity. Lean MASLD accounts for approximately 5.1% of the global population and is more prevalent in Asia, where genetic predispositions such as *PNPLA3* and *TM6SF2* polymorphisms, visceral obesity, and high-carbohydrate dietary patterns are key risk factors. Lean MASLD is also associated with significant liver and non-liver complications, as well as increased all-cause mortality risk. Therefore, lean MASLD may pose a significant challenge for practitioners.

## Introduction

Metabolic dysfunction-associated steatotic liver disease (MASLD) has emerged as a leading cause of chronic liver disease globally, affecting up to 30% of the global population (1). However, its prevalence can rise to nearly 50% among individuals with type 2 diabetes mellitus (T2DM) (1, 2). Formerly known as non-alcoholic fatty liver disease (NAFLD), the nomenclature was updated to MAFLD (metabolic dysfunction-associated fatty liver disease) in 2020 to better emphasize the important role of metabolic risk factors, the complex pathophysiology in disease development, and to establish the diagnosis based on positive criteria rather than exclusion (3, 4). Moreover, MAFLD terminology has been proposed to be changed to MASLD. The term fatty liver disease has been changed to steatotic liver disease (SLD) to reduce the stigma and discrimination associated with obesity, which can negatively impact quality of life and contribute to social and emotional burdens (5–8). While MASLD is traditionally associated with obesity and metabolic syndrome, a substantial subset of MASLD also occurs in individuals with a normal body mass index (BMI) and is known as lean MASLD (9).

Lean MASLD is estimated to account for approximately 5-13% of the worldwide population (10, 11). It presents a unique clinical challenge, as clinicians may overlook the condition in the absence of overt obesity, thus leading to underdiagnosis (12). Lean MASLD individuals still experience significant disease progression, including advanced fibrosis, cirrhosis, and increased cardiovascular risk, similar to their obese counterparts (13). Emerging studies have shown that lean MASLD may represent a distinct clinical entity and therefore requires further investigation of its pathophysiology, clinical progression, and associated risk factors. Understanding these aspects is crucial for identifying the underlying disease mechanisms, predicting outcomes, and optimizing management strategies tailored to this specific subgroup (13, 14).

Interestingly, MASLD also demonstrates regional differences between Asian and Western populations. Lean MASLD is notably more prevalent in Asia, where its higher occurrence may be attributed to the combination of genetic predispositions – such as *PNPLA3* polymorphism – and regional lifestyle factors, including carbohydrate-rich diets and lower physical activity levels (15). In contrast, lean MASLD in Western populations is often associated with body composition differences, visceral adiposity, and high-fat diet (9, 16).

Therefore, this narrative review aims to explore the global landscape of lean MASLD, with a particular focus on the key differences between Asian and Western populations. We also highlights the impact of lean MASLD compared to non-lean MASLD.

## The MASLD and lean MASLD definition

Steatotic liver disease is defined as the accumulation of excess fat in the liver, exceeding 5% of the organ’s weight. Classically, SLD was divided into two categories based on the etiology of the steatosis and amount of alcohol intake: MASLD and ALD (17). MASLD is a novel clinical term for fatty liver disease, which emphasizes the role of metabolic dysfunction. Previously, the diagnosis of NAFLD was established after excluding other causes of liver disease. In contrast, MASLD is diagnosed by fulfilling a set of positive clinical criteria (3, 4). Therefore, MASLD can be defined as SLD in the presence of at least one cardiometabolic risk factor (**Table 1**), with no other identifiable etiology, and without significant alcohol intake (weekly alcohol consumption not exceeding 140 grams for females or 210 grams for males; daily alcohol consumption not exceeding 20 grams for females or 30 grams for males). Lean MASLD can be diagnosed in individuals with lean or normal body weight (BMI < 25 kg/m^2^ in Caucasians or BMI < 23 kg/m^2^ in Asians), who nevertheless fulfill the other MASLD diagnostic criteria (3, 5, 6).

**Table 1:** Adult cardiometabolic criteria for diagnosing MASLD (6).

- BMI > 25 kg/m^2^ in Caucasian or BMI > 23 kg/m2 in Asian OR Waist circumference >102 cm in Caucasian men and > 88 cm in Caucasian women OR ethnicity adjusted equivalent
- HbA1c ≥ 5.7% OR fasting blood glucose >100 mg/dl OR 2 hour post prandial ≥140 mg/dl OR type 2 diabetes OR on treatment for type 2 diabetes
- Elevated blood pressure (≥ 130/85 mmHg) OR on blood pressure lowering therapy
- Elevated triglycerides level (≥ 150 mg/dl) OR on lipid lowering therapy
- Low HDL cholesterol (< 40 mg/dl in men and < 50 mg/dl in women) OR on lipid lowering therapy

Hepatic steatosis can be confirmed by imaging modalities (ultrasonography, transient elastography, computed tomography [CT] scan, magnetic resonance imaging [MRI], magnetic resonance elastography), blood biomarkers or scores, or by liver histopathological examination (5, 6). Several blood biomarkers and scores are being extensively studied as non-invasive methods for diagnosing and predicting the severity of MASLD (18–20).

MASLD may progress to metabolic dysfunction-associated steatohepatitis (MASH), which is characterized by the presence of lobular inflammation and hepatocyte ballooning, with or without fibrosis. MASH, a more severe form of MASLD, develops as a result of prolonged inflammation and hepatic injury induced by lipotoxicity, oxidative stress, gut dysbiosis, and metabolic dysregulation. MASH is associated with accelerated fibrogenesis and may eventually progress to cirrhosis. Studies have shown that early stages of MASLD and MASH may be reversible (18–21). Cirrhosis is defined as the late stage of any chronic liver disease, characterized by extensive fibrosis and regenerative nodules. Cirrhosis can lead to significant morbidity and mortality and may progress to hepatocellular carcinoma (HCC). Therefore, liver transplantation remains the definitive treatment, and ongoing research remains focused on identifying strategies to reverse hepatic fibrosis and cirrhosi**s** (22, 23).

## Global epidemiology of MASLD

The burden of MASLD has been steadily increasing in recent years, driven by the rising prevalence of obesity and other components of metabolic syndrome (MetS). This upward trend reflects the growing global epidemic of metabolic disorders, which pose a significant public health challenge. According to the World Health Organization (WHO), the prevalence of adult obesity has quadrupled in recent decades, with approximately 2.5 billion adults classified as overweight and 890 million classified as obese by 2022, accounting for 43% and 16% of the adult population, respectively. With 1 in 8 people worldwide living with obesity, the condition has become an epidemic with significant global health impacts (24).

Similarly, the prevalence of T2DM, a major risk factor for MASLD, has also risen sharply. The global population affected by diabetes increased from 200 million in 1990 to 830 million in 2022, underscoring the significant rise in metabolic dysfunction worldwide (25). The burden of metabolic syndrome is equally alarming. A recent systematic review reported that the global prevalence of MetS ranged from 12.5% to 31.4%, depending on the diagnostic criteria used. Among its components, central obesity was the most prevalent, affecting 45% of individuals, followed by elevated blood pressure (42.6%), low HDL cholesterol levels (40.2%), elevated triglycerides (28.9%), and impaired fasting glucose (24.6%) (26).

These increasing trends in MASLD risk factors have resulted in MASLD affecting 25–30% of the global population. It is estimated that as many as 1.7 billion individuals are affected by MASLD worldwide (27). The global prevalence of MASLD has increased significantly, rising from 25.3% between 1990 and 2006 to 38.2% between 2016 and 2019, reflecting a nearly 50% rise over the past three decades (1). The global prevalence of MASLD showed significant regional variations, with the highest rates observed in the Middle East (32%), followed by South America (31%) and Asia (27%). Meanwhile, the United States of America (USA) and Europe reported rates of 24% and 23%, respectively (2). Miao et al. reported similar MASLD prevalence rates with 44.4% in Latin America, 36.5% in the Middle East and North Africa (MENA), 33.8% in South Asia, 33.1% in Southeast Asia, 31.2% in North America and Australia, 29.7% in East Asia, 28.0% in the Asia Pacific, and 25.1% in Western Europe (1). The prevalence of MASLD has been reported to reach up to 65% in individuals with T2DM (28). A recent cohort study in the USA showed that 54% of MASLD patients were obese (29). In China, the prevalence of MASLD was reported to be 59.8% among obese individuals, 27.4% among those who were overweight, and 4.0% among individuals with normal weight (30).

## Comparison of epidemiology of lean MASLD in Asia and the West

**Figure 1:**
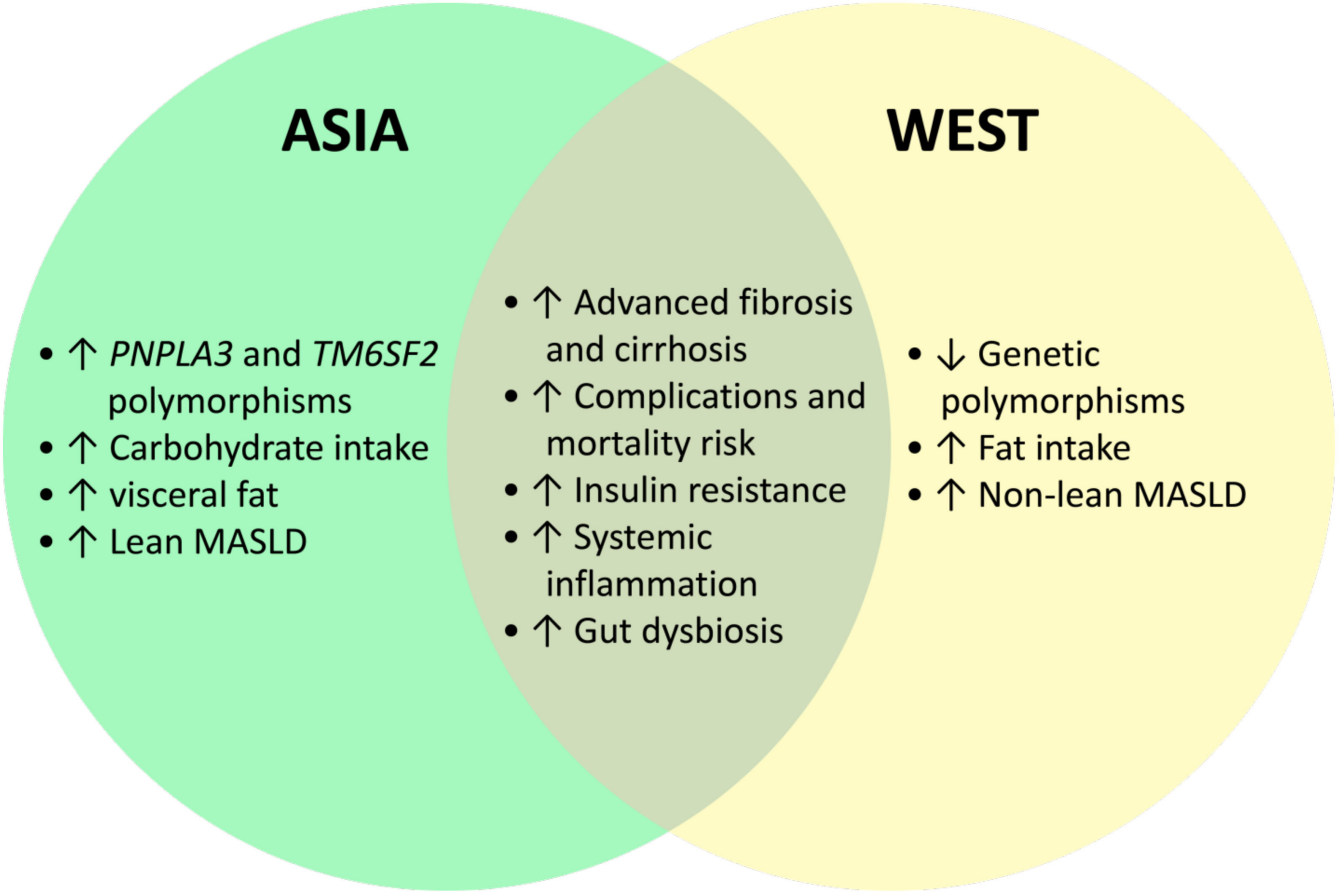
Comparison of MASLD characteristics in Asia and West populations.

The worldwide prevalence of lean MASLD is estimated at approximately 5.1% in the general population. Among the MASLD patients, 19.2% were lean. Lean MASLD is more common in Asia (30, 31). A systematic review showed that the prevalence of lean MASLD was 12% in Asian countries compared with 9.2% in Western countries (32). Another study showed that the prevalence of lean MASLD in Asia was 14.55%, contributing to one-third of all MASLD cases (11). According to an international registry study in Asia, approximately one-fifth of MASLD patients were non-obese (33). A study in China reported a prevalence of 8.98% for lean MASLD (34). There are several factors that are thought to be linked to higher lean MASLD prevalence in Asia compared to the West, such as genetic predisposition, dietary patterns, physical activity levels, and sarcopenia (**Figure 1**) (35).

## Genetic predisposition role in Asian lean MASLD

Several genetic variants associated with fat metabolism and insulin resistance play a pivotal role in the development of hepatic steatosis in lean individuals, even in the absence of obesity. Among these, patatin-like phospholipase domain-containing 3 (*PNPLA3*) and *TM6SF2* polymorphisms are the most extensively studied in Asian populations as determining factor for MASLD development (36).

The *PNPLA3* gene polymorphism is linked to altered lipid metabolism and ketogenesis, especially in the liver. *PNPLA3* variant increases hepatic ketogenesis and intrahepatic lipolysis, enhances beta-oxidation, and alters the hepatic mitochondrial redox state. Furthermore, the *PNPLA3* variant also decreases hepatic *de novo* lipogenesis. Altogether, these mechanisms lead to hepatic mitochondrial dysfunction and represent major risk factors for MASLD (37). *PNPLA3* is also associated with increased hepatic triglycerides due to decreased triglyceride hydrolysis (38). *PNPLA3* variant is more commonly found in Asia. A study in Korea showed that 20-48.4% of MASLD patients had *PNPLA3* polymorphism with the G allele regarded as a high-risk allele (39). A study also showed that *PNPLA3* rs738409 is associated with lean MASLD development (40). A study in Hong Kong showed PNPLA3 rs738409 was an independent factor in the development of lean MASLD (40). The impact of the G allele of *PNPLA3* rs738409 in non-obese MASLD was also demonstrated in a Japanese study (41). A recent genome-wide association study (GWAS) by Hsu et al. in a Taiwanese Han Chinese population identified the *PNPLA3* rs9625962 (C allele) variant as the most significant genetic determinant associated with the occurrence of lean MASLD, independent of triglyceride levels. The *SAMM50* variant was also reported to have a contributory role and may act synergistically with *PNPLA3* in predisposing individuals to lean MASLD (42). A study by Lu et al. in Taiwan found that the *PNPLA3* rs12483959 variant was independently associated with a threefold increased risk of developing lean MASLD, followed by the *SAMM50* rs3761472 variant, which conferred a 2.9-fold higher risk (43). A study conducted in Hong Kong demonstrated that the *PNPLA3* rs738409 GG genotype was more frequently observed in lean MASLD patients compared to overweight or obese individuals and was also associated with higher intrahepatic triglyceride content (44). A study regarding the *PNPLA3* as a significant risk factor for MASLD has been conducted in Kariadi Hospital, Indonesia. That study included 152 patients, which consisting of 80 MASLD patients and 72 controls. We found significant association between *PNPLA3* rs738409 variant and MASLD diagnosis (p=0.009, OR 2.52, CI 95% 1.25-5.07). Further genotype analysis revealed that the G allele was associated with a higher proportion of NASH compared to simple steatosis. This finding may be attributed to *PNPLA3* encoding adiponutrin, a protein responsible for triacylglycerol hydrolysis, which leads to triglyceride accumulation in the liver (45). The pathogenic effect of the *PNPLA3* variant appears to be independent of metabolic parameters such as serum lipid profile, blood glucose level, and body mass index (BMI), thereby reinforcing the role of genetic variation as a key driver in the development of lean MASLD (46**).** The *PNPLA3* variant is also associated with a more aggressive disease course and poorer prognosis in patients with MASLD. Pennisi et al. also found that *PNPLA3* rs738409 C>G variant, which is mainly responsible for lean MASLD, is associated with a higher risk of fibrosis progression in MASLD (47). In a longitudinal study by Grimaudo et al. with a median follow-up of five years, the *PNPLA3* C>G variant was found to be independently associated with a twofold increased risk of hepatic decompensation, a 2.66-fold higher risk of developing HCC, and a 3.64-fold greater risk of liver-related mortality (48).

The *TM6SF2* gene variant was also implicated in a higher prevalence of lean MASLD in Asian countries (30). A study in South India found that *TM6SF2* was significantly associated with 1.9 to 2.7-fold higher risk of developing MASLD (49). The role of *TM6SF2* rs58542926 polymorphism in MASLD development was also demonstrated in the Chinese population (50). A study in Korea involving more than 6, 900 participants reported that over 30% of lean MASLD patients carried the *TM6SF2* (rs58542926) heterozygous minor alleles (CT or TT) (51). A 2022 meta-analysis concluded that the *TM6SF2* rs58542926 variant was associated with a 1.6-fold increased risk of developing MASLD and higher AST levels, despite being linked to lower serum lipid concentrations (52). *TM6SF2*, located on chromosome 19, might alter VLDL secretion and triglyceride accumulation. This in turn leads to fatty liver and liver dysfunction (53).

Several other genetic polymorphisms were also linked to lean MASLD development, especially in Asia, such as membrane bound O-acyltransferase 7 domain (*MBOAT7*) gene, lysophospholipase like-1 (*LYPLAL1)*, liver fatty acid-binding protein (*FABP*), glucokinase regulator (*GCKR*), and others (54–56). Serum miR-4488 has emerged as a promising biomarker for diagnosing and predicting lean MASLD in Asian individuals (57). Altogether, these gene polymorphisms may explain the increased lean MASLD prevalence among Asians.

## Gut microbiome variation role in Asian lean MASLD

The gut microbiome has been implicated in MASLD development. The variation in gut microbiome composition may lead to distinct phenotypes of MASLD among Western and Asian populations. Microbiome composition is closely related to dietary factors (58). Dysbiosis may lead to insulin resistance, altered bile acid composition, increased endogenous ethanol and choline production (18). Gut dysbiosis is associated with increased gut permeability and inflammation, leading to microbial translocation and a chronic inflammatory state due to endotoxemia. The gut microbiome also plays a vital role in the production of short-chain fatty acids (SCFAs). Altogether, these multiple mechanisms lead to the development of MASLD (59).

A study in Thailand reported distinct gut microbiome composition and diversity in lean MASLD patients. Chuaypen et al. found increased populations of *Escherichia-Shigella* and decreased population of *Lachnospira* and *Subdoligranulum* in lean MASLD patients compared to the obese MASLD group (60). Another study in China showed that lean MASLD patients had lower diversity in the gut microbiome, reduced *Firmicutes* population, and increased *Bacteroidetes* populations. Among these, *Firmicutes* were responsible for producing SCFAs which are important in maintaining gut barrier integrity (61). Lee et al. found that *Ruminococcaceae* and *Veillonellaceae* were related to fibrosis progression in lean Asian MASLD patients (62).

Alterations in secondary bile acids and SCFAs have been observed in lean MASLD, indicating gut–liver axis dysregulation, which is likely associated with underlying gut dysbiosis. A recent study by Haag et al. highlighted the differences between SCFAs composition in lean vs. obese MASLD. In lean MASLD, metabolomic profiling revealed higher concentrations of isobutyrate, methionine sulfoxide, propionate, and phosphatidylcholines. In contrast, obese MASLD was distinguished by elevated sarcosine levels alongside reduced lysine and asymmetric dimethylarginine. Elevated propionate and isobutyrate levels in lean MASLD patients may be attributed to dysbiosis and dietary factor. Propionate and isobutyrate induce hepatic steatosis through different pathways (63). A study by Chen et al. demonstrated elevated levels of secondary bile acids and fibroblast growth factor 19 (FGF19), and decreased 7-alpha-hydroxy-4-cholesten-3-one (C4), which were linked to a higher degree of steatohepatitis and fibrosis in lean MASLD patients (30). Altogether, the bile acids and SCFAs may be related to liver and intestinal farnesoid X-receptor (FXR) signaling pathway inhibition. FXR plays a crucial role in controlling insulin sensitivity, glucose and lipid metabolism, cell growth, intestinal barrier integrity, inflammatory pathways, and fibrogenesis. In the liver, FXR activation suppresses *de novo* lipogenesis, regulates lipid transport, enhances fatty acid oxidation, promotes triglyceride hydrolysis, and induces FGF21 expression, collectively exerting a protective effect against MASH (64). Recent multi-omics profiling studies have demonstrated distinct compositional differences between lean and obese MASLD, further supporting the role of gut-liver axis dysregulation in the lean phenotype MASLD. *Streptococcus* spp. were found to be more abundant among individuals with lean MASLD, leading to several altered metagenomic pathways, including enhanced lipid synthesis (reflected by increased PWY-6270 activity), upregulation of pyruvate-related pathways and acetone formation from pyruvate, as well as dysregulation of pyrimidine metabolism and other metabolic processes. Moreover, gut microbiome–derived metabolites also help define MASLD subtypes, with polyamine metabolites being significantly elevated in patients with lean MASLD (65). Anirvan et al. also demonstrated that distinct gut microbiome profiles, together with genetic polymorphisms, play a significant role in determining the lean versus obese MASLD phenotype (66).

Therefore, studies investigating probiotic/synbiotic supplementation for the management of MASLD are currently being extensively conducted. Identifying the presence of gut dysbiosis, followed by comprehensive microbiome profiling and targeted restoration of microbial balance, may represent a promising future strategy (67). Alam et al. demonstrated a significant improvement in the controlled attenuation parameter among patients with lean MASLD after six months of probiotic supplementation, irrespective of changes in BMI (68). A meta-analysis conducted in 2024 demonstrated that synbiotic supplementation was linked with improvements in liver inflammation parameters, such as AST and ALT, inflammatory markers (e.g., TNF-α), lipid profile, and insulin resistance in patients with MASLD (69).

## Lifestyle role in Asian lean MASLD

### Dietary factors

The high prevalence of carbohydrate-rich diets in many Asian countries significantly impacts the gut microbiota composition. Diets high in refined sugars and low in fiber can promote the proliferation of gut bacteria that metabolize carbohydrates into SCFAs and other metabolites, contributing to insulin resistance and hepatic steatosis. These dietary patterns, combined with reduced physical activity in urbanized settings, exacerbate gut dysbiosis and metabolic dysfunction, particularly in lean individuals (70).

Carbohydrates and fats undergo distinct metabolic pathways after intestinal absorption. Dietary fats, primarily in the form of chylomicrons, are transported to extrahepatic tissues through the lymphatic system, thereby bypassing the liver. In contrast, carbohydrates are delivered directly to the liver, where excessive intake stimulates glycogen synthesis and *de novo* lipogenesis (DNL), converting surplus glucose into fatty acids. These newly synthesized lipids may be stored within hepatocytes, secreted as very-low-density lipoprotein (VLDL) particles, or oxidized for energy during fasting. Carbohydrate intake, especially glucose, stimulates insulin secretion, which in turn activates sterol regulatory element-binding protein (SREBP). This activation leads to the upregulation of hepatic lipogenic genes, resulting in an increase in hepatic DNL. Another carbohydrate, such as fructose has been identified as a more potent stimulator of hepatic DNL signaling (71). A high-fructose diet has been associated with increased hepatic lipid peroxidation and upregulation of hepatic glucose transporter type 5 (GLUT5), along with the activation of multiple proinflammatory pathways that collectively contribute to the development of steatohepatitis (72).

Several animal model studies have demonstrated that methionine- and choline-deficient diets (MCD) as well as high-fructose diets can induce the development of lean MASLD (73). MCD feeding is associated with a reduction in *Bifidobacterium* and an increase in *Bacteroides*, resulting in gut dysbiosis and altered SCFAs production. In this model, hepatic steatosis develops after approximately two weeks, while NASH with liver fibrosis becomes evident after about four weeks of MCD administration (74). Zhang et al. found that a high-carbohydrate diet induced a greater degree of hepatic fat accumulation without causing obesity, compared with mice fed a high-cholesterol high-calorie diet (75). Another animal model study demonstrated that feeding mice a very high-carbohydrate diet for 17 weeks resulted in significant triacylglycerol accumulation in the liver (76).

Kang et al. found that, in a Korean population with and without MASLD, a high-carbohydrate diet was associated with insulin resistance and alterations in gut microbiota composition, characterized by an increase in *Enterobacteriaceae* and a decrease in *Ruminococcaceae* and *Veillonellaceae*. The abundance of *Enterobacteriaceae* showed a positive correlation with SREBF2 (sterol regulatory element binding factor-2) activation, a key regulator of DNL, thereby promoting hepatic lipid accumulation and steatosis (77). A study in the Japanese population showed that high intake of carbohydrates and rice was associated with increased MASLD prevalence, but not with bread and noodles (78). A study at Kariadi Hospital, Indonesia, showed that high carbohydrate intake was associated with a 7.8-fold higher risk for developing MASLD in Indonesian people (79). Another study in Korea showed that high consumption of carbohydrates was associated with 1.63-1.88-fold higher risk for MASLD (70). Another hypothesis suggests that high intake of sugar and simple carbohydrates may interact with the *PNPLA3* genetic variant, leading to hepatic fat accumulation in lean individuals (80). These findings highlight the differences in dietary patterns between Asia and the West, where Western diets are typically high in fat, leading to obesity (81, 82).

### Sarcopenia and visceral obesity

Several studies have associated sarcopenia with the development of lean MASLD (83, 84). A study in Korea showed that low muscle mass was associated with a 1.8-fold increased risk for developing lean MASLD (84). A meta-analysis also reported a 1.54-fold higher risk of MASLD in sarcopenic patients (85). The proposed mechanism linking sarcopenia and MASLD development involves myosteatosis and insulin resistance. Increased cell injury and inflammation, decreased glycogen synthesis and storage, increased *de novo* lipogenesis, oxidative stress, lipotoxicity, and increased proteolysis are shared mechanisms between sarcopenic muscle and MASLD development (86). This finding underscores that obesity is not always the cause of MASLD, and distinguishes lean MASLD as a distinct entity, especially among sarcopenic patients.

Various studies have shown that Asians have a higher rate of visceral obesity than Caucasians (87). The common method for determining ideal body weight, such as calculating the BMI, may fail to identify visceral obesity. However, it is generally agreed that visceral fat is a key indicator of MASLD development and plays a crucial role in defining the lean MASLD subgroup. Central obesity is associated with metabolic abnormalities caused by the active secretion of pro-inflammatory adipokines by visceral fat. Visceral fat is also associated with reduced hepatic insulin extraction, hyperinsulinemia, increased gluconeogenesis, and the production of triglyceride-rich lipoproteins. Visceral fat also promotes free fatty acid accumulation in the liver and the release of inflammatory cytokines, leading to a hit to liver cells due to a chronic inflammatory state. Protein and fat metabolism are also altered in patients with visceral obesity (88–91). A study by Purnomo et al. showed that hypoadiponectinemia was an independent risk factor for developing MASLD (92). Hypoadiponectinemia is more strongly associated with visceral fat compared than subcutaneous fat accumulation (93). A study in Indonesia showed visceral fat deposition was a significant risk factor for developing MASLD (*OR* = 50.7, CI95% 6.16-418.09) and was associated with severe MASLD (*OR* = 6.6, CI95% 1.17-37.78) (79).

## Impact of lean MASLD compared to obese MASLD in Asia

Lean MASLD is often underrecognized in clinical practice; however, it should be regarded as a clinically significant entity, as it confers a higher risk for several complications compared to obese MASLD.

A meta-analysis by Wongtrakul et al., which included 94, 181 MASLD patients showed that lean MASLD individuals had a 1.6-fold higher risk of all-cause mortality compared than non-lean MASLD individuals (94). A study in Korea showed that lean MASLD patients had a 2.34-fold increased risk of mortality compared to obese MASLD or MASLD with diabetes (95).

Another meta-analysis by Ha et al. showed that lean MASLD patients had a 1.88-fold increased risk of liver-related mortality compared than non-lean NAFLD (96). Lean MASLD patients also showed an increased risk of developing accelerated fibrosis and cirrhosis. Nabi et al. found that lean MASLD individuals were a 1.26-fold more likely to develop advanced fibrosis (97). A retrospective study conducted in the USA between 1999 and 2016 reported a higher prevalence of advanced fibrosis among lean MASLD individuals (98). A long-term study by Danpanichkul et al. demonstrated that individuals with lean MASLD had a higher risk of developing hepatocellular carcinoma (HCC) (99). Despite having a normal BMI, patients with lean MASLD tend to have a higher risk of cardiac complications, such as acute coronary syndromes and metabolic syndrome (18, 100, 101). Lean MASLD is also associated with a higher risks of developing non-liver-associated complications such as diabetes mellitus, hypertension, and new-onset cardiovascular disease (102).

## Recommendation for future directions

Based on all the findings presented above, we can conclude that lean MASLD should be recognized as a distinct clinical entity, different from “typical” MASLD (non-lean MASLD) as it confers higher risks for liver and non-liver-related complications, as well as higher mortality risk. Lean MASLD also tends to be underrecognized in clinical practice. BMI calculation alone may not be sufficient during the examination of MASLD. Visceral fat and metabolic dysfunction assessment should be performed in all lean patients to screen the possibility of lean MASLD. We summarize the different characteristics of obese MASLD and lean MASLD in **Table 2**.

**Table 2:** Key differences in between lean and obese MASLD characteristics.

Characteristics	Lean MASLD	Obese MASLD
Body composition	Normal or low BMI (<23 kg/m² in Asians, <25 kg/m² in Caucasians); often increased visceral fat and decreased skeletal muscle mass (sarcopenia)	Elevated BMI; generalized obesity with increased subcutaneous and visceral adiposity
Epidemiology	More common in Asian populations; accounts for 10–20% of MASLD cases	More prevalent in Western populations; represents majority of MASLD cases
Pathogenesis	Predominantly due to genetic predisposition (PNPLA3, TM6SF2 variants), visceral adiposity, sarcopenia, gut dysbiosis, and high-carbohydrate diet	Primarily driven by obesity-related insulin resistance, high-fat diet, and metabolic syndrome
Metabolic profile	May have mild or absent metabolic syndrome features; insulin resistance and dyslipidemia may still be present	Frequently associated with metabolic syndrome, diabetes mellitus, hypertension, and dyslipidemia
Genetic predisposition	PNPLA3 and TM6SF2 variants strongly associated; higher prevalence in Asians	PNPLA3 and GCKR variants contribute but less dominant compared to metabolic factors
Gut microbiota	Reduced diversity, lower Firmicutes, increased Bacteroidetes; associated with endotoxemia and inflammation	Altered microbiota composition, often linked to high-fat diet and obesity
Dietary pattern	High-carbohydrate, low-fiber diet; refined starch and rice common in Asia	High-fat, high-calorie Western diet; excess saturated fat intake
Sarcopenia	Common; loss of muscle mass contributes to insulin resistance and hepatic fat accumulation	Less frequent; excess muscle lipid infiltration possible
Clinical course and prognosis	Often underdiagnosed; higher risk of fibrosis progression, cardiovascular events, and all-cause mortality despite normal BMI	Well-recognized; Complications are also contributed due to underlying obesity

Although increasing evidence has expanded our understanding of lean MASLD, most studies remain limited by cross-sectional design, small sample size, and heterogeneity in diagnostic criteria. Differences in BMI cutoffs between Asian and Western populations complicate direct comparisons of prevalence and outcomes. Moreover, the majority of data originate from tertiary centers, which may not represent the general population. Integrative analyses that combine genomic, metabolomic, and microbiome data remains limited, and longitudinal studies examining disease progression, fibrosis, and hepatocellular carcinoma risk in lean individuals are sparse. Future research should focus on standardized definitions, population-based cohort studies, and mechanistic trials exploring therapeutic strategies tailored for lean MASLD.
